# Synthesis, crystal structure, Hirshfeld surface analysis and DFT calculations of the coordination compound tetra­aqua­bis­{2-[(5-methyl-1,3,4-thia­diazol-2-yl)sulfan­yl]acetato-κ*O*}cobalt(II)

**DOI:** 10.1107/S2056989024011939

**Published:** 2025-01-01

**Authors:** Ekaterina Kinshakova, Batirbay Torambetov, Simranjeet Kaur, Jamshid Ashurov, Shakhnoza Kadirova

**Affiliations:** a National University of Uzbekistan named after Mirzo Ulugbek 4 University St Tashkent 100174 Uzbekistan; b Physical and Material Chemistry Division CSIR-National Chemical Laboratory,Pune 411008 India; c Academy of Scientific and Innovative Research (AcSIR) Ghaziabad 201002 India; d Institute of Bioorganic Chemistry, Academy of Sciences of Uzbekistan, M. Ulugbek St, 83, Tashkent, 100125, Uzbekistan; Vienna University of Technology, Austria

**Keywords:** crystal structure, cobalt(II), 1,3,4-thia­diazole, hydrogen bonding, Hirshfeld surface analysis, DFT calculation

## Abstract

A novel coordination compound, [Co(*L*)_2_(H_2_O)_4_], was synthesized from aqueous solutions of Co(NO_3_)_2_ and the ligand 2-[(5-methyl-1,3,4-thia­diazol-2-yl)sulfan­yl]acetic acid (H*L*, C_5_H_6_N_2_O_2_S_2_). In the monoclinic crystals (space group *P*2_1_/*c*), the cobalt(II) ion is located about a centre of symmetry and is octa­hedrally coordinated by two *L^−^* anions in a monodentate fashion through carboxyl O atoms and by four water mol­ecules. A relatively strong hydrogen bond between one of the water mol­ecules and the non-coordinating carboxyl­ate O atom consolidates the conformation. In the crystal, inter­molecular hydrogen bonds lead to the formation of a complex tri-periodic structure. Hirshfeld surface analysis revealed that 30.1% of the inter­molecular inter­actions are from H⋯H contacts and 20.8% are from N⋯H/H⋯N contacts. DFT calculations were performed to assess the stability and chemical reactivity of the compound by determining the energy differences between the HOMO and LUMO.

## Abstract 2

The mol­ecular and crystal structure of the tetra­aqua­bis­{2-[(5-methyl-1,3,4-thia­diazol-2-yl)sulfan­yl]acetato}­cobalt(II) complex were studied and Hirshfeld surfaces and fingerprint plots were generated to investigate the various inter­molecular inter­actions.

## 1. Chemical context

1,3,4-Thia­diazole derivatives are versatile compounds with significant applications in various fields, notably as ligands in the formation of metal complexes (Frija *et al.*, 2016 ▸). Their ability to coordinate metal ions through multiple donor atoms allows for the creation of stable and diverse complexes (Atashov *et al.*, 2024 ▸; Lavrenova *et al.*, 2023 ▸; Serbest *et al.*, 2008 ▸), which can be tailored for specific applications in medicine (Masaryk *et al.*, 2022 ▸; Patil *et al.*, 2020 ▸; Karcz *et al.*, 2020 ▸), agriculture (Smaili *et al.*, 2017 ▸; Chandra *et al.*, 2015 ▸) or materials science (Bawazeer *et al.*, 2020 ▸; Karasmani *et al.*, 2018 ▸; Wang *et al.*, 2012 ▸).
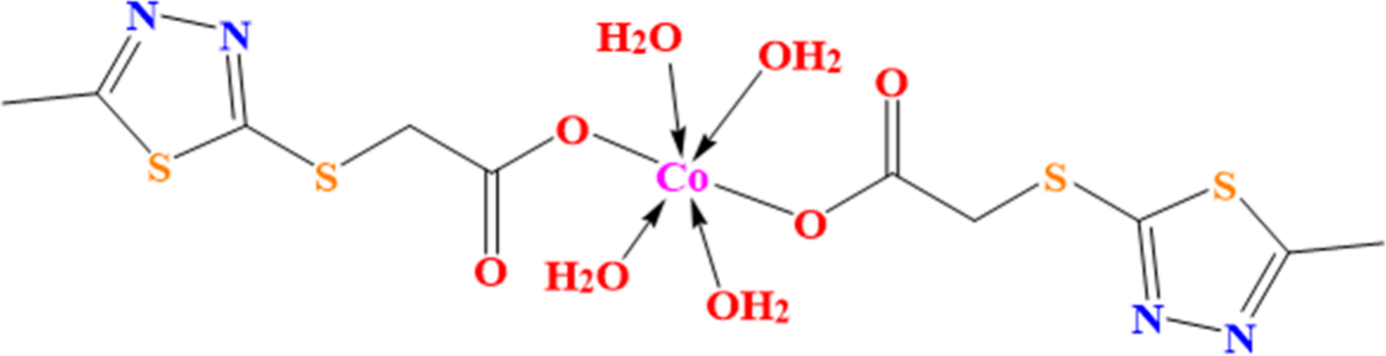


[(5-Methyl-1,3,4-thia­diazol-2-yl)sulfan­yl]acetic acid (H*L*) is a derivative of 5-methyl-1,3,4-thia­diazole-2-thiol by S-alkyl­ation. It is a sulfur-containing carb­oxy­lic acid, which is widely used due to its unique properties. It is a non-toxic and water-soluble compound in which the substituent is located in the form of a pharmacophore, which can lead to higher reactivity and biological activity through complexation. In this context, we report here on the title coordination compound [Co(*L*)_2_(H_2_O)_4_].

## 2. Structural commentary

**Figure 1:**
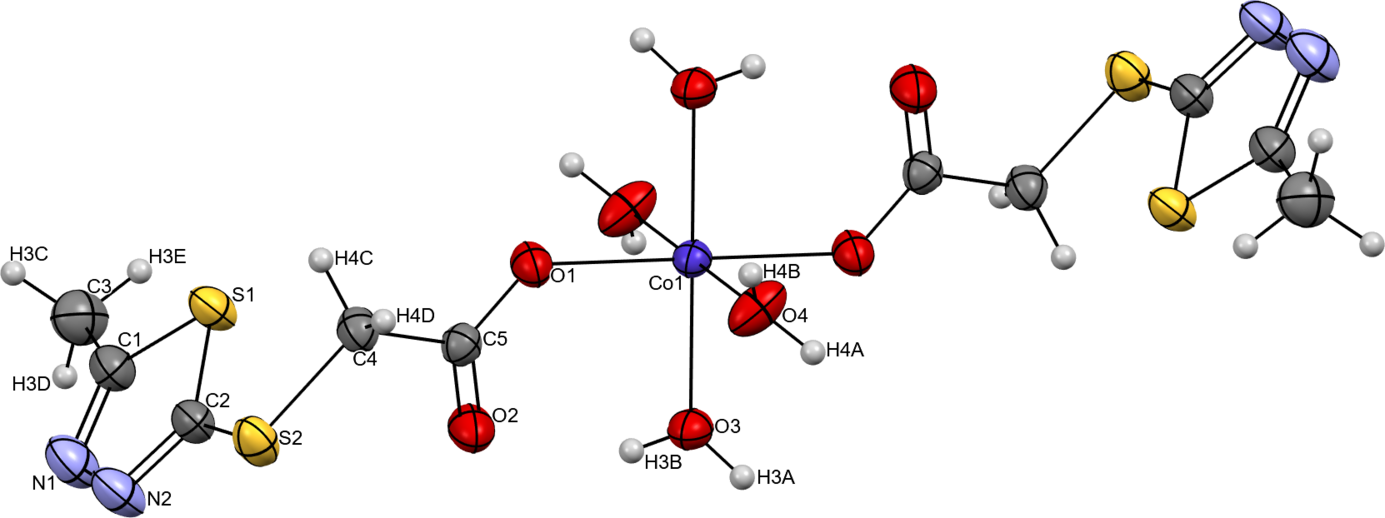
The mol­ecular structure of the title complex [Co(*L*)_2_(H_2_O)_4_]. Displacement ellipsoids are shown at the 20% probability level. Non-labelled atoms are generated by inversion symmetry [symmetry operation: 1 − *x*, 1 − *y*, 1 − *z*].

The mol­ecular structure of [Co(*L*)_2_(H_2_O)_4_] is shown in Fig. 1 ▸. The asymmetric unit comprises half a mol­ecule of the complex, with the Co^II^ atom located about a centre of symmetry. The Co^II^ atom exhibits a slightly distorted octa­hedral coordination environment formed by carboxyl­ate and water O atoms. The carboxyl­ate group coordinates monodentately through O1, O1^i^ together with water O atoms O4 and O4^i^ in the equatorial plane [symmetry code: (i) –*x* + 1, –*y* + 1, –*z* + 1], whereas the two water O atoms O3 and O3^i^ are in axial positions. The corresponding distances are listed in Table 1 ▸. The *cis*-bond angles in the coordination polyhedron vary from 84.80 (8) to 95.20 (8)°. Bond lengths and angles of the 5-methyl-1,3,4-thia­diazole-2-thiol­ate ligand are similar to the standard values observed in similar structures (see section 6). The positions of the ligands allow for the formation of one rather strong hydrogen bond between a water mol­ecule (O3—H3*B*) and the non-coordinating carboxyl­ate O4 atom (Table 2 ▸), which is shorter than the stated distance (2.85 Å) in liquid water (Eisenberg & Kauzmann, 2005 ▸). This hydrogen bond leads to a six-membered ring motif with designation *S*(6) (Etter, 1990 ▸; Etter *et al.*, 1990 ▸; Grabowski, 2020 ▸). Moreover, crystal-packing effects result in the C4—H4*C*⋯S distances (Table 2 ▸) being smaller than the sum of the van der Waals radii, and the existing short intra­molecular contacts can be considered from a geometrical and topological point of view as a weak hydrogen bond contributing to the overall cohesion of the mol­ecular conformation (Fargher *et al.*, 2022 ▸; Domagała *et al.*, 2003 ▸; Surange *et al.*, 1997 ▸).

## 3. Supra­molecular features and energy framework calculations

**Figure 2:**
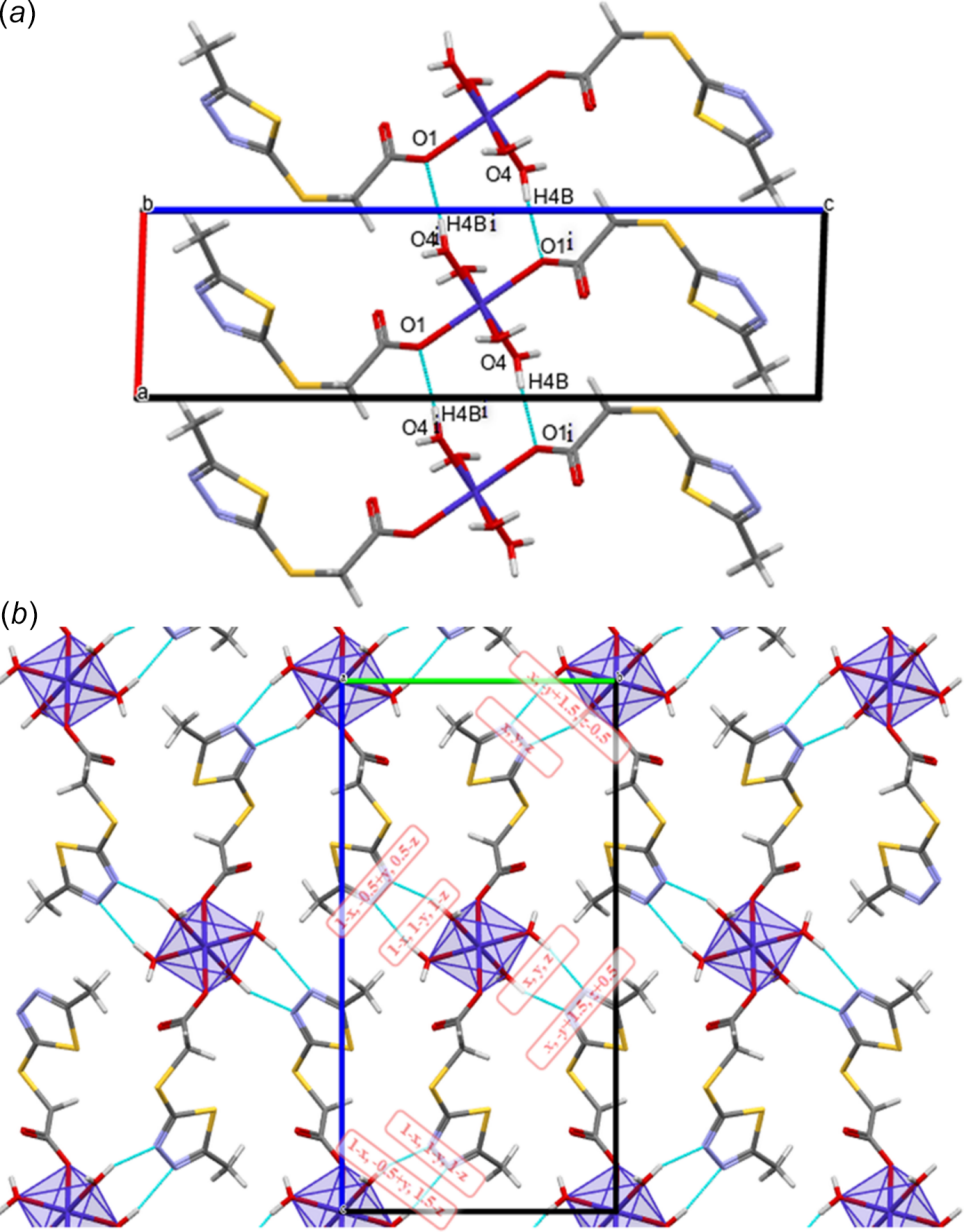
Overview of hydrogen-bonding inter­actions and the crystal packing: (*a*) Inter­actions along the *a* axis; (*b*) inter­actions along the *b* and *c* axes, highlighting the O3—H3*A*⋯N1 and O4—H4*A*⋯N2 hydrogen bonds (as per symmetry operators).

In the crystal structure of [Co(*L*)_2_(H_2_O)_4_], further hydrogen bonds are observed (Table 2 ▸). Neighboring cobalt complexes are connected parallel to the *a* axis by O4—H4*B*⋯O1^i^ and O4^i^—H4*B*^i^ ⋯O1 inter­actions; the corresponding Co1⋯Co1^i^ distance is 5.195 Å (Fig. 2 ▸*a*). Neighboring central atoms are located at distances of 10.035 (1) and 18.909 (1) Å, respectively, along the *b-* and *c*-axis directions, and cohesion in the crystal is achieved due to the O3—H3*A*⋯N1 and O4—H4*A*⋯N2 hydrogen bonds (Fig. 2 ▸*b*).

**Figure 3:**
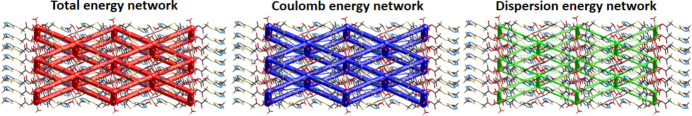
Calculated inter­action energies in the title compound. The thickness of the tubes correspond to the value of the energy, revealing strong inter­actions along the crystallographic *a*-axis direction (the largest values are represented here). The total energy framework (in red) and its two main components, dispersion (in green) and Coulombic energy (in blue), shown for a cluster around a reference mol­ecule, also exhibit stronger inter­actions along the crystallographic *a*-axis direction.

**Figure 4:**
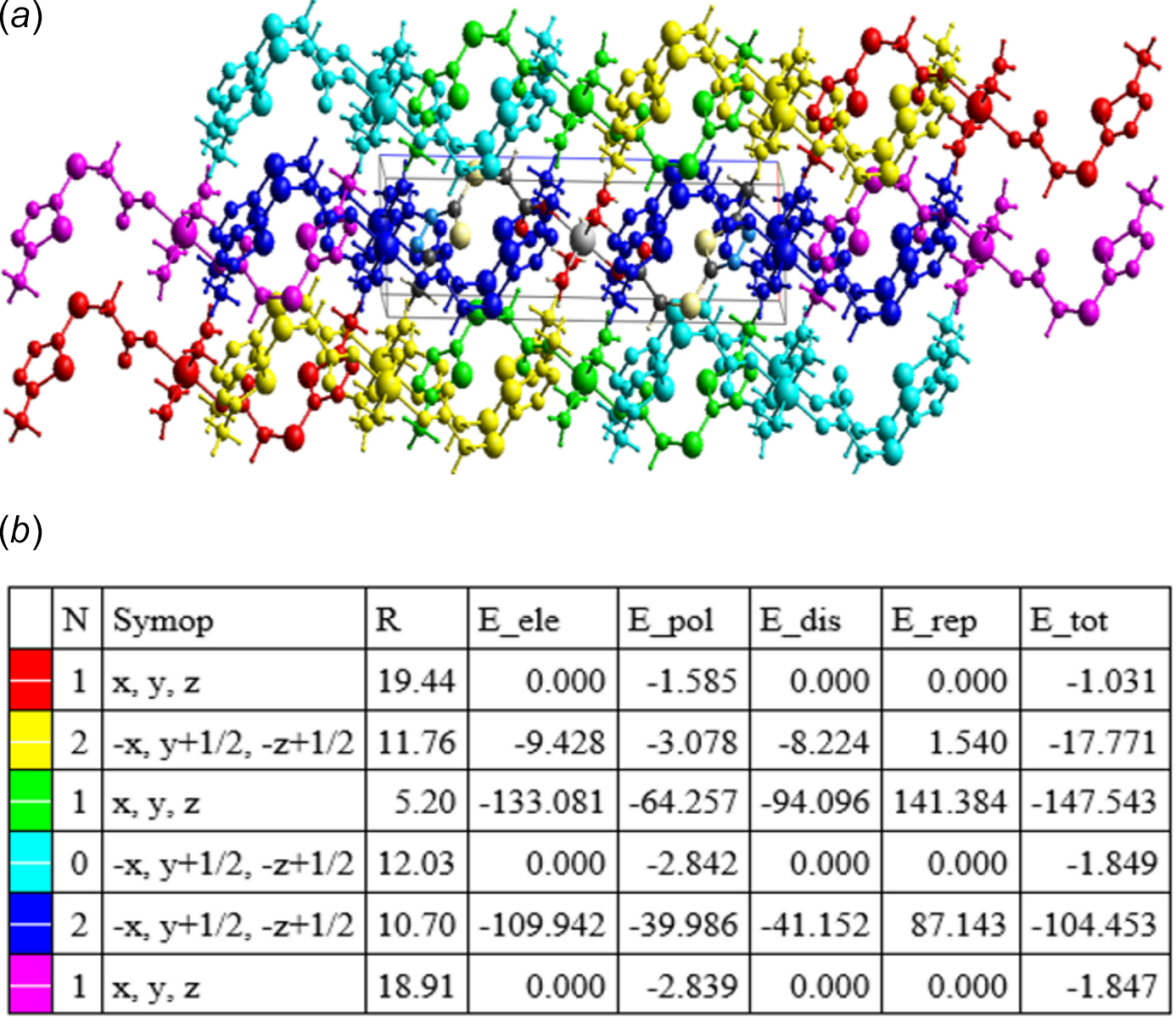
(*a*) Energy framework diagram for compound [Co(*L*)_2_(H_2_O)_4_]; (*b*) the color-coded inter­action mapping within 3.8 Å of the center mol­ecule (gray) calculated with the CE—HF⋯HF/3–21 G model (*R* is the distance between mol­ecular centroids (main atomic position) in Å, all inter­action energies in kJ mol^−1^).

The inter­action energies of the hydrogen-bonding system were calculated using the HF method (HF/3-21G) in *CrystalExplorer* (Spackman *et al.*, 2021 ▸). The result is represented graphically in Fig. 3 ▸, showing the total energy (*E*_tot_), which is the sum of the Coulombic (*E*_ele_), polar (*E*_pol_), dispersion (*E*_dis_) and repulsive (*E*_rep_) contributions. The four energy components were scaled for the total energy calculation (*E*_tot_ = 1.019*E*_ele_ + 0.651*E*_pol_ + 0.901*E*_dis_ + 0.811*E*_rep_). The inter­action energies were investigated for a 3.8 Å cluster around the reference mol­ecule and are depicted in Fig. 4 ▸*a* as framework energy diagrams. The components of the inter­action energies (*E*), symmetry operations concerning the reference mol­ecule (Symop), the centroid-to-centroid distances between the reference mol­ecule and inter­acting mol­ecules (*R*), and the number of pair(s) of inter­acting mol­ecules to the reference mol­ecule (*N*) are listed in Fig. 4 ▸*b*. The total inter­action energy is −274.5 kJ mol^−1^, involving the electrostatic (–257.2 kJ mol^−1^), polarization (–74.6 kJ mol^−1^), dispersion (–129.3 kJ mol^−1^), and repulsion (186.6 kJ mol^−1^) energies. The main attractive inter­actions (Coulombic, dispersion and the sum total energy) show a stronger bonding effect along the crystallographic *a*-axis direction.

## 4. Hirshfeld surface analysis

**Figure 5:**
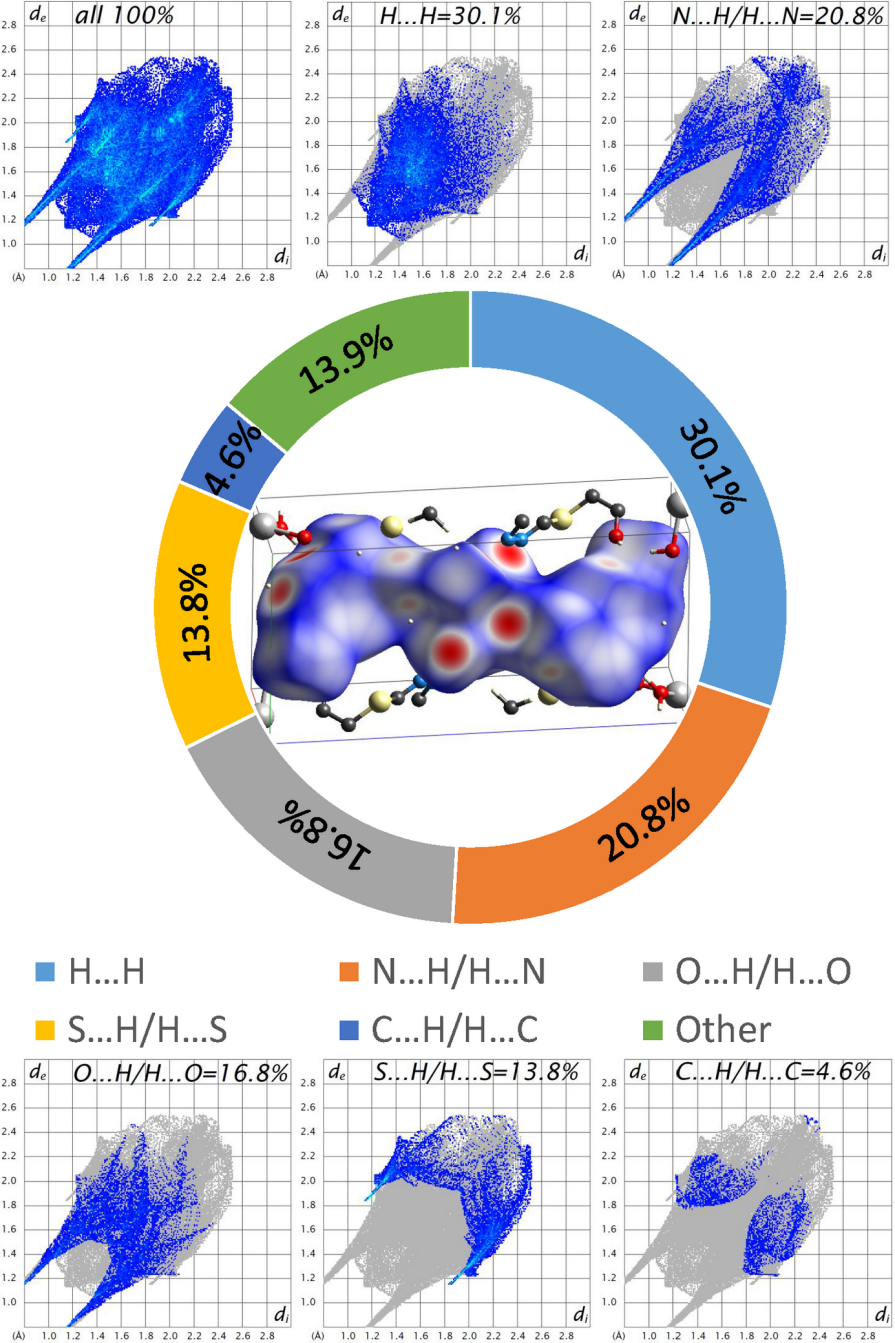
View of the three-dimensional Hirshfeld surface of [Co(*L*)_2_(H_2_O)_4_] (central part). The full two-dimensional fingerprint plots for the title complex, showing all inter­actions and delineated into separate inter­actions with the percentage contribution of various inter­atomic contacts occurring in the crystal are shown at the top and bottom.

To further investigate the inter­molecular inter­actions present in the title compound, a Hirshfeld surface (HS) analysis was performed, and the two-dimensional fingerprint plots were generated with *CrystalExplorer* (Spackman *et al.*, 2021 ▸). Fig. 5 ▸ shows the three-dimensional Hirshfeld surface of the complex plotted over *d*_norm_ (normalized contact distance). The hydrogen-bonding inter­actions given in Table 2 ▸ play a key role in the mol­ecular packing of the complex.

The overall two-dimensional fingerprint plot and those delineated into inter­atomic inter­actions are given in Fig. 5 ▸. The HS analysis shows that the most important contributions are from H⋯H, N⋯H/H⋯N, O⋯H/H⋯O and S⋯H/H⋯S contacts, while other contributions (C⋯H/H⋯C, S⋯O/O⋯S, S⋯C/C⋯S and S⋯N/N⋯S) are considered as minor contacts. The percentage contributions of the various inter­atomic contacts occurring in the crystal are also shown in Fig. 5 ▸.

## 5. Density functional theory (DFT) calculations

The mol­ecular structure of the complex was optimized in the gas phase by the B3LYP (Lee *et al.*, 1988 ▸) DFT method using the LanL2dz basis set (Grimme, 2006 ▸) for the Co atom and 6-311G (d,p) basis set (de Castro & Jorge, 1998 ▸) for non-metal atoms. Calculations were conducted using the *Gaussian09* program (Frisch *et al.*, 2009 ▸) to evaluate the stability of the compound and its chemical reactivity by determining the HOMO–LUMO (highest occupied mol­ecular orbital - lowest unoccupied mol­ecular orbital) energy differences, the ionization potential (*I*), the affinity electronics (*A*), the electrophilicity index (ω), the chemical potential (μ), the hardness (η) and the softness (*S*). The optimized potential surface mol­ecular electrostatic potential (MEP) was also determined to characterize the effects of various substituent groups. Additionally, an analysis was performed to identify regions of electron richness and deficiency.

**Figure 6:**
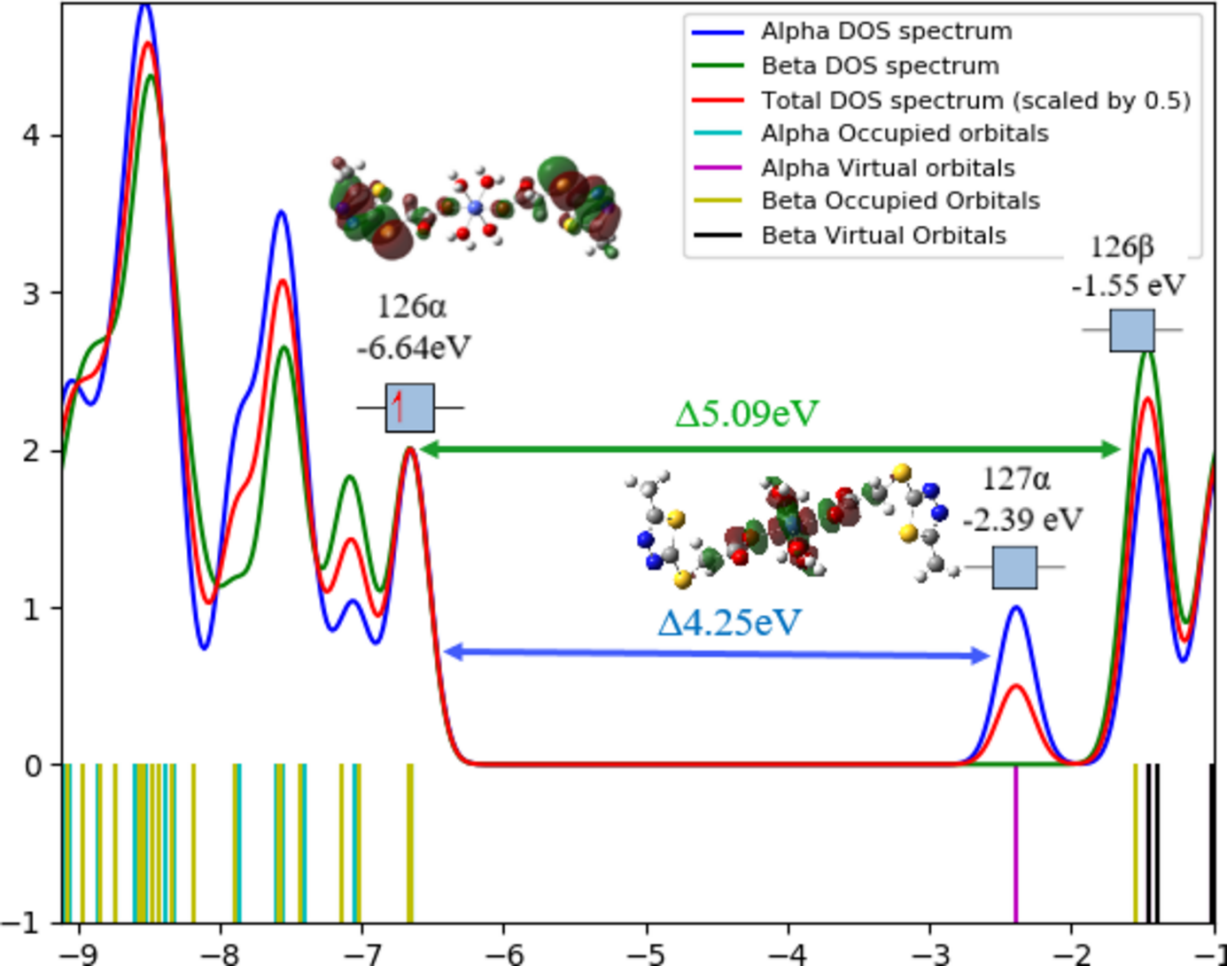
DOS spectrum, SOMO and LUMO energies and energy gap in the title complex.

It is well known that for a closed-shell mol­ecule (all electrons are paired) the HOMO–LUMO energy gap is related to its stability. The same argument is used for open-shell systems (unrestricted calculation). In this case, it is taken into account that the energies of both α- and β-spin orbitals designate the orbital with the highest energy as SOMO (singly occupied mol­ecular orbital). Similarly, the LUMO is defined among both α and β, and the corresponding gap is considered as the SOMO–LUMO gap (Abella *et al.*, 2021 ▸). Fig. 6 ▸ shows the SOMO and LUMO of the title cobalt complex, as well as the DOS (density of states) spectrum displaying the group contributions to the mol­ecular orbitals and the calculation of the density of states (*Gauss-Sum 3.0*). The DOS spectra were obtained by combining the mol­ecular orbital information with the extraction from *Gaussian* (O’boyle *et al.*, 2008 ▸). The descriptors of the reactivity of the complex derived from the electronic properties of the specified mol­ecule (Padmanabhan *et al.*, 2007 ▸; Hekim & Pekdemir, 2022 ▸) based on the energies of the HOMO (SOMO) and LUMO orbitals, are shown in Table 3 ▸.

**Figure 7:**
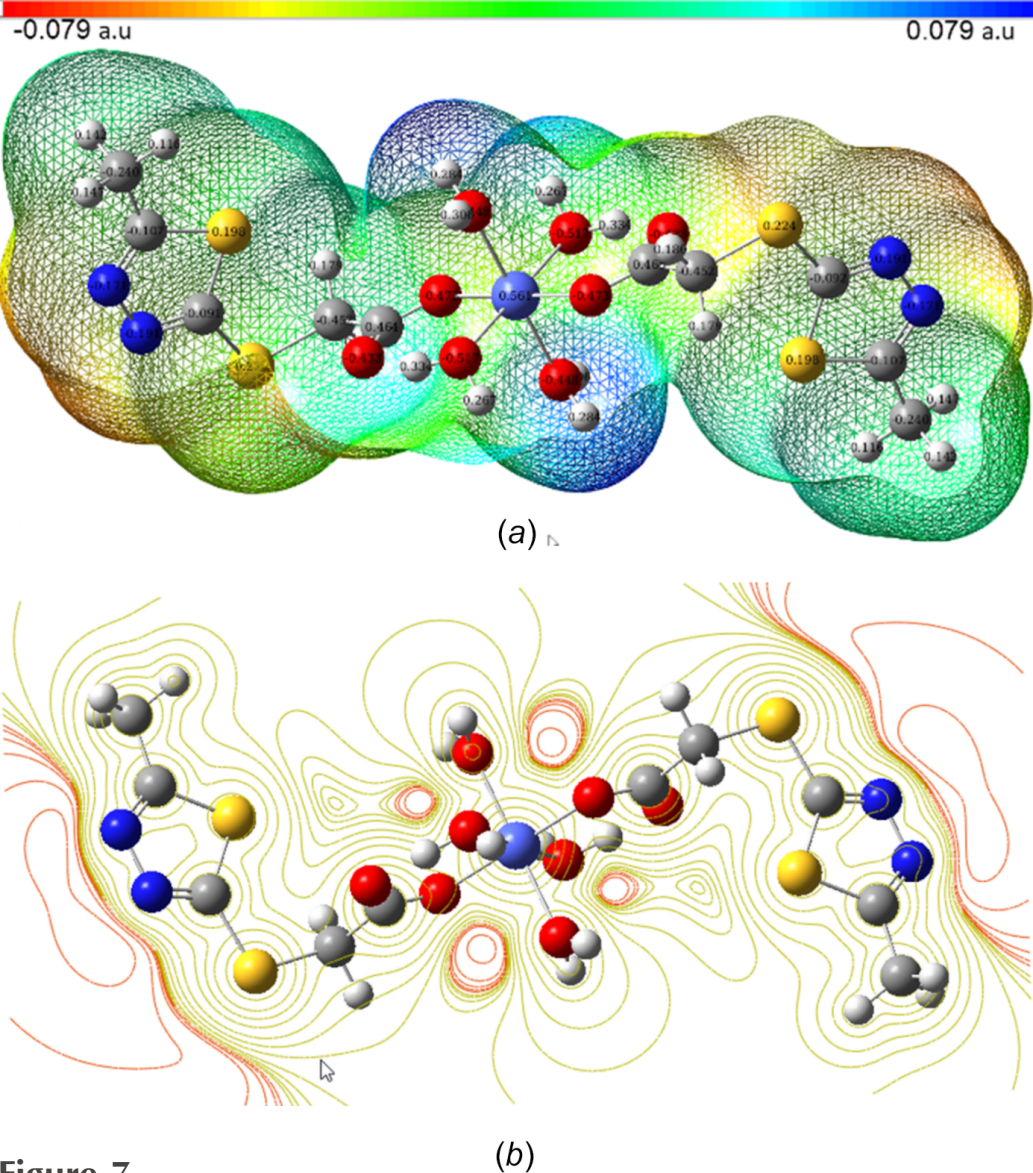
The total electron density surface and the surface of the contour in the form of two dimensional surface curves of [Co(*L*)_2_(H_2_O)_4_].

The electrophilic and nucleophilic nature of the inter­actions, as well as hydrogen bonds, can be explained using the mol­ecular electrostatic potential (MEP), which is related to the electron density. The total electron density surface and the surface of the contour in the form of two-dimensional surface curves of the title complex is given in Fig. 7 ▸, which has color codes from red to blue, representing negative to positive potential distributions. The optimum electrophilic reaction zones are localized on N atoms, the maximum positive regions that determine nucleophilic reactions are concentrated at the hydrogen atoms of the water mol­ecules. These locations provide insight into the regions of the mol­ecule that engage in non-covalent inter­actions.

## 6. Database survey

A survey of the Cambridge Structural Database (CSD, Version 5.45, last updated March 2024; Groom *et al.*, 2016 ▸) indicates that crystal structures have been reported for complexes of 2-methyl-1,3,4-thia­diazole derivatives with several metal ions, including Co, Ni, Cu, Zn, Ru, Ag, Pd, Cd, Sn, Pr, Nd, Pt, Hg, and Bi. Notably, there are ten reported metal complex structures featuring 2-[(5-methyl-1,3,4-thia­diazol-2-yl)thio]­acetate (denoted as *L*), specifically: DEKGAE, DEKGEI (Pan & Zheng, 2017 ▸); ICOVED, ICOVIH (Pan, 2011 ▸); QAFRAS, QAFREW (Pan *et al.*, 2010 ▸) and UNENAD, UNENEH, UNENIL, UNENOR (Ma *et al.*, 2010 ▸). In these structures, *L* coordinates to the metal ions through the oxygen atom of the carb­oxy­lic group. Notably, in the UNENEH structure, *L* does not directly coordinate to the Co^II^ cation. It is inter­esting to note that the title compound differs from the UNENAD structure by the absence of one water mol­ecule.

## 7. Synthesis and crystallization

A solution of Co(NO_3_)_2_·6H_2_O (0.291 g, 0.1 mmol) in C_2_H_5_OH (3 ml) was added to a solution of H*L* (0.38 g, 0.2 mmol) in C_2_H_5_OH/H_2_O (5 ml, 1:1), the pH of which was adjusted to 7.0 with dilute sodium hydroxide (1 mol/l). The mixture was stirred at room temperature for 5 h to obtain a clear solution, which was then filtered. Slow evaporation of the filtrate after two weeks gave pink single crystals in the form of blocks suitable for X-ray diffraction. Yield: 0.367 g (72%).

## 8. Refinement

Crystal data, data collection and structure refinement details are summarized in Table 4 ▸. All hydrogen atoms were located in difference-Fourier maps and reﬁned using an isotropic approximation. The water hydrogen atoms were constrained to an ideal geometry with distances fixed at 0.87 (4) Å and *U*_iso_(H) = 1.5*U*_eq_(O). Two reflections (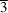 10 9; 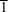 11 11) were omitted from the refinement..

## Supplementary Material

Crystal structure: contains datablock(s) I. DOI: 10.1107/S2056989024011939/wm5739sup1.cif

Structure factors: contains datablock(s) I. DOI: 10.1107/S2056989024011939/wm5739Isup3.hkl

CCDC reference: 2408680

Additional supporting information:  crystallographic information; 3D view; checkCIF report

## Figures and Tables

**Table 1:** Selected bond lengths (Å)

Co1—O1	2.088 (2)	Co1—O4	2.036 (2)
Co1—O3	2.146 (2)		

**Table 2:** Hydrogen-bond geometry (Å, °)

*D*—H⋯*A*	*D*—H	H⋯*A*	*D*⋯*A*	*D*—H⋯*A*
O3—H3*B*⋯O2	0.87 (2)	1.91 (3)	2.700 (3)	151 (5)
C4—H4*C*⋯S1	0.97	2.79	3.181 (3)	105
O4—H4*A*⋯N2^i^	0.85	1.95	2.764 (4)	160
O4—H4*B*⋯O1^ii^	0.85	1.91	2.757 (3)	172
O3—H3*A*⋯N1^i^	0.84 (5)	2.17 (5)	2.979 (4)	162 (5)

Symmetry codes: (i) 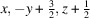; (ii) 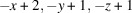.

**Table 3:** Global reactivity indices

*E* SOMO (eV)	−6.64
*E* LUMO (eV)	−2.39
Δ*E*	4.25
*I*	6.64
*A*	2.39
χ	4.515
η	2.125
μ	−4.515
S	0.235
ω	0.235

**Table 4:** Experimental details

Crystal data	
Chemical formula	[Co(C_5_H_5_N_2_O_2_S_2_)_2_(H_2_O)_4_]
*M*_r_	509.45
Crystal system, space group	Monoclinic, *P*2_1_/*c*
Temperature (K)	293
*a*, *b*, *c* (Å)	5.1950 (2), 10.0347 (3), 18.9090 (6)
β (°)	91.892 (3)
*V* (Å^3^)	985.19 (6)
*Z*	2
Radiation type	Cu *K*α
μ (mm^−1^)	11.23
Crystal size (mm)	0.08 × 0.06 × 0.04

Data collection	
Diffractometer	Xcalibur, Ruby
Absorption correction	Multi-scan (*CrysAlis PRO*; Rigaku OD, 2021 ▸)
*T*_min_, *T*_max_	0.808, 1.000
No. of measured, independent and observed [*I* > 2σ(*I*)] reflections	6554, 2023, 1770
*R*_int_	0.055
(sin θ/λ)_max_ (Å^−1^)	0.630

Refinement	
*R*[*F*^2^ > 2σ(*F*^2^)], *wR*(*F*^2^), *S*	0.038, 0.098, 1.06
No. of reflections	2023
No. of parameters	137
No. of restraints	1
H-atom treatment	H atoms treated by a mixture of independent and constrained refinement
Δρ_max_, Δρ_min_ (e Å^−3^)	0.34, −0.27

Computer programs: *CrysAlis PRO* (Rigaku OD, 2021 ▸), *SHELXT* (Sheldrick, 2015*a* ▸), *SHELXL* (Sheldrick, 2015*b* ▸), *OLEX2* (Dolomanov *et al.*, 2009 ▸) and *publCIF* (Westrip, 2010 ▸).

## supplementary crystallographic information

### Tetraaquabis{2-[(5-methyl-1,3,4-thiadiazol-2-yl)sulfanyl]acetato-κO}cobalt(II) Crystal data

**Table:**

[Co(C_5_H_5_N_2_O_2_S_2_)_2_(H_2_O)_4_]	*F*(000) = 522
*M**_r_* = 509.45	*D*_x_ = 1.717 Mg m^−^^3^
Monoclinic, *P*2_1_/*c*	Cu *K*α radiation, λ = 1.54184 Å
*a* = 5.1950 (2) Å	Cell parameters from 2568 reflections
*b* = 10.0347 (3) Å	θ = 4.4–74.9°
*c* = 18.9090 (6) Å	µ = 11.23 mm^−^^1^
β = 91.892 (3)°	*T* = 293 K
*V* = 985.19 (6) Å^3^	Block, pink
*Z* = 2	0.08 × 0.06 × 0.04 mm

### Tetraaquabis{2-[(5-methyl-1,3,4-thiadiazol-2-yl)sulfanyl]acetato-κO}cobalt(II) Data collection

**Table:**

Xcalibur, Ruby diffractometer	2023 independent reflections
Radiation source: fine-focus sealed X-ray tube, Enhance (Cu) X-ray Source	1770 reflections with *I* > 2σ(*I*)
Graphite monochromator	*R*_int_ = 0.055
Detector resolution: 10.2576 pixels mm^-1^	θ_max_ = 76.1°, θ_min_ = 4.7°
ω scans	*h* = −6→5
Absorption correction: multi-scan (CrysAlisPro; Rigaku OD, 2021)	*k* = −12→7
*T*_min_ = 0.808, *T*_max_ = 1.000	*l* = −22→23
6554 measured reflections	

### Tetraaquabis{2-[(5-methyl-1,3,4-thiadiazol-2-yl)sulfanyl]acetato-κO}cobalt(II) Refinement

**Table:**

Refinement on *F*^2^	Hydrogen site location: mixed
Least-squares matrix: full	H atoms treated by a mixture of independent and constrained refinement
*R*[*F*^2^ > 2σ(*F*^2^)] = 0.038	*w* = 1/[σ^2^(*F*_o_^2^) + (0.0423*P*)^2^ + 0.2531*P*] where *P* = (*F*_o_^2^ + 2*F*_c_^2^)/3
*wR*(*F*^2^) = 0.098	(Δ/σ)_max_ < 0.001
*S* = 1.06	Δρ_max_ = 0.34 e Å^−^^3^
2023 reflections	Δρ_min_ = −0.27 e Å^−^^3^
137 parameters	Extinction correction: SHELXL (Sheldrick, 2015b), Fc^*^=kFc[1+0.001xFc^2^λ^3^/sin(2θ)]^-1/4^
1 restraint	Extinction coefficient: 0.0100 (6)
Primary atom site location: dual	

### Tetraaquabis{2-[(5-methyl-1,3,4-thiadiazol-2-yl)sulfanyl]acetato-κO}cobalt(II) Special details

**Table:**

Geometry. All esds (except the esd in the dihedral angle between two l.s. planes) are estimated using the full covariance matrix. The cell esds are taken into account individually in the estimation of esds in distances, angles and torsion angles; correlations between esds in cell parameters are only used when they are defined by crystal symmetry. An approximate (isotropic) treatment of cell esds is used for estimating esds involving l.s. planes.

### Tetraaquabis{2-[(5-methyl-1,3,4-thiadiazol-2-yl)sulfanyl]acetato-κO}cobalt(II) Fractional atomic coordinates and isotropic or equivalent isotropic displacement parameters (Å^2^)

**Table:**

	*x*	*y*	*z*	*U*_iso_*/*U*_eq_	
Co1	0.500000	0.500000	0.500000	0.0225 (2)	
S2	0.93444 (16)	0.67263 (8)	0.23436 (4)	0.0363 (2)	
S1	0.51630 (16)	0.46582 (8)	0.19232 (4)	0.0352 (2)	
O1	0.7298 (4)	0.4971 (2)	0.41151 (11)	0.0301 (5)	
O3	0.3120 (5)	0.6845 (2)	0.47316 (12)	0.0319 (5)	
O2	0.5470 (5)	0.6628 (2)	0.34843 (12)	0.0381 (5)	
O4	0.7849 (4)	0.5994 (2)	0.55445 (14)	0.0403 (6)	
H4A	0.754101	0.661162	0.583781	0.060*	
H4B	0.939850	0.576984	0.564102	0.060*	
N2	0.6147 (6)	0.6755 (3)	0.12306 (16)	0.0439 (7)	
N1	0.4211 (7)	0.6115 (3)	0.08461 (16)	0.0466 (7)	
C5	0.7135 (6)	0.5770 (3)	0.35950 (15)	0.0261 (6)	
C4	0.9347 (6)	0.5598 (3)	0.30843 (16)	0.0308 (6)	
H4C	0.929270	0.469381	0.290307	0.037*	
H4D	1.096227	0.570265	0.335059	0.037*	
C2	0.6821 (6)	0.6126 (3)	0.18058 (16)	0.0314 (6)	
C1	0.3501 (7)	0.5011 (3)	0.11352 (17)	0.0350 (7)	
C3	0.1401 (8)	0.4148 (4)	0.0839 (2)	0.0464 (8)	
H3C	0.208492	0.355386	0.049477	0.070*	
H3D	0.008604	0.469362	0.061893	0.070*	
H3E	0.067607	0.363860	0.121388	0.070*	
H3A	0.311 (9)	0.750 (5)	0.501 (2)	0.061 (14)*	
H3B	0.383 (9)	0.707 (5)	0.4341 (17)	0.070 (15)*	

### Tetraaquabis{2-[(5-methyl-1,3,4-thiadiazol-2-yl)sulfanyl]acetato-κO}cobalt(II) Atomic displacement parameters (Å^2^)

**Table:**

	*U*^11^	*U*^22^	*U*^33^	*U*^12^	*U*^13^	*U*^23^
Co1	0.0219 (3)	0.0233 (3)	0.0222 (3)	0.0038 (2)	−0.0007 (2)	−0.0002 (2)
S2	0.0405 (5)	0.0368 (4)	0.0316 (4)	−0.0094 (3)	0.0025 (3)	0.0057 (3)
S1	0.0430 (5)	0.0323 (4)	0.0301 (4)	−0.0059 (3)	−0.0032 (3)	0.0096 (3)
O1	0.0298 (11)	0.0348 (11)	0.0259 (10)	0.0063 (8)	0.0038 (8)	0.0037 (8)
O3	0.0374 (13)	0.0277 (10)	0.0304 (11)	0.0073 (9)	−0.0013 (9)	−0.0001 (8)
O2	0.0392 (13)	0.0426 (12)	0.0326 (11)	0.0156 (10)	0.0032 (10)	0.0060 (9)
O4	0.0248 (11)	0.0418 (13)	0.0534 (15)	0.0074 (9)	−0.0121 (10)	−0.0217 (10)
N2	0.0510 (18)	0.0427 (16)	0.0375 (15)	−0.0075 (13)	−0.0077 (13)	0.0156 (12)
N1	0.0571 (19)	0.0473 (17)	0.0348 (15)	−0.0039 (14)	−0.0077 (14)	0.0123 (13)
C5	0.0263 (14)	0.0275 (13)	0.0242 (13)	−0.0011 (11)	−0.0008 (11)	−0.0012 (10)
C4	0.0299 (15)	0.0350 (15)	0.0276 (14)	0.0044 (12)	0.0018 (11)	0.0022 (11)
C2	0.0373 (17)	0.0302 (14)	0.0270 (14)	0.0016 (12)	0.0036 (12)	0.0062 (11)
C1	0.0422 (19)	0.0347 (16)	0.0281 (15)	0.0056 (13)	0.0010 (13)	0.0047 (11)
C3	0.048 (2)	0.0464 (19)	0.0437 (19)	−0.0003 (16)	−0.0115 (16)	0.0015 (15)

### Tetraaquabis{2-[(5-methyl-1,3,4-thiadiazol-2-yl)sulfanyl]acetato-κO}cobalt(II) Geometric parameters (Å, º)

**Table:**

Co1—O1^i^	2.088 (2)	O2—C5	1.233 (4)
Co1—O1	2.088 (2)	O4—H4A	0.8501
Co1—O3	2.146 (2)	O4—H4B	0.8500
Co1—O3^i^	2.146 (2)	N2—N1	1.380 (4)
Co1—O4	2.036 (2)	N2—C2	1.296 (4)
Co1—O4^i^	2.036 (2)	N1—C1	1.295 (4)
S2—C4	1.801 (3)	C5—C4	1.535 (4)
S2—C2	1.740 (3)	C4—H4C	0.9700
S1—C2	1.724 (3)	C4—H4D	0.9700
S1—C1	1.734 (3)	C1—C3	1.488 (5)
O1—C5	1.270 (3)	C3—H3C	0.9600
O3—H3A	0.84 (5)	C3—H3D	0.9600
O3—H3B	0.868 (19)	C3—H3E	0.9600
			
O1^i^—Co1—O1	180.0	C2—N2—N1	112.8 (3)
O1—Co1—O3	95.20 (8)	C1—N1—N2	112.9 (3)
O1^i^—Co1—O3^i^	95.20 (8)	O1—C5—C4	112.6 (2)
O1—Co1—O3^i^	84.80 (8)	O2—C5—O1	127.0 (3)
O1^i^—Co1—O3	84.80 (8)	O2—C5—C4	120.5 (3)
O3—Co1—O3^i^	180.0	S2—C4—H4C	108.3
O4—Co1—O1^i^	90.76 (10)	S2—C4—H4D	108.3
O4^i^—Co1—O1	90.76 (10)	C5—C4—S2	115.9 (2)
O4^i^—Co1—O1^i^	89.24 (10)	C5—C4—H4C	108.3
O4—Co1—O1	89.24 (10)	C5—C4—H4D	108.3
O4—Co1—O3	90.83 (9)	H4C—C4—H4D	107.4
O4—Co1—O3^i^	89.17 (9)	S1—C2—S2	126.18 (18)
O4^i^—Co1—O3	89.17 (9)	N2—C2—S2	120.0 (2)
O4^i^—Co1—O3^i^	90.83 (9)	N2—C2—S1	113.7 (3)
O4—Co1—O4^i^	180.00 (11)	N1—C1—S1	113.4 (3)
C2—S2—C4	102.60 (15)	N1—C1—C3	123.7 (3)
C2—S1—C1	87.22 (15)	C3—C1—S1	122.8 (2)
C5—O1—Co1	125.90 (19)	C1—C3—H3C	109.5
Co1—O3—H3A	123 (3)	C1—C3—H3D	109.5
Co1—O3—H3B	103 (3)	C1—C3—H3E	109.5
H3A—O3—H3B	109 (4)	H3C—C3—H3D	109.5
Co1—O4—H4A	122.5	H3C—C3—H3E	109.5
Co1—O4—H4B	130.3	H3D—C3—H3E	109.5
H4A—O4—H4B	104.5		
			
Co1—O1—C5—O2	−6.9 (4)	C4—S2—C2—S1	−8.8 (3)
Co1—O1—C5—C4	171.97 (18)	C4—S2—C2—N2	176.0 (3)
O1—C5—C4—S2	−178.0 (2)	C2—S2—C4—C5	−73.2 (2)
O2—C5—C4—S2	0.9 (4)	C2—S1—C1—N1	0.1 (3)
N2—N1—C1—S1	0.4 (4)	C2—S1—C1—C3	−177.7 (3)
N2—N1—C1—C3	178.3 (3)	C2—N2—N1—C1	−1.0 (5)
N1—N2—C2—S2	176.8 (2)	C1—S1—C2—S2	−176.1 (2)
N1—N2—C2—S1	1.1 (4)	C1—S1—C2—N2	−0.7 (3)

Symmetry code: (i) −*x*+1, −*y*+1, −*z*+1.

### Tetraaquabis{2-[(5-methyl-1,3,4-thiadiazol-2-yl)sulfanyl]acetato-κO}cobalt(II) Hydrogen-bond geometry (Å, º)

**Table:**

*D*—H···*A*	*D*—H	H···*A*	*D*···*A*	*D*—H···*A*
O3—H3*B*···O2	0.87 (2)	1.91 (3)	2.700 (3)	151 (5)
C4—H4*C*···S1	0.97	2.79	3.181 (3)	105
O4—H4*A*···N2^ii^	0.85	1.95	2.764 (4)	160
O4—H4*B*···O1^iii^	0.85	1.91	2.757 (3)	172
O3—H3*A*···N1^ii^	0.84 (5)	2.17 (5)	2.979 (4)	162 (5)

Symmetry codes: (ii) *x*, −*y*+3/2, *z*+1/2; (iii) −*x*+2, −*y*+1, −*z*+1.
